# Structural power and the evolution of collective fairness in social networks

**DOI:** 10.1371/journal.pone.0175687

**Published:** 2017-04-14

**Authors:** Fernando P. Santos, Jorge M. Pacheco, Ana Paiva, Francisco C. Santos

**Affiliations:** 1 INESC-ID and Instituto Superior Técnico, Universidade de Lisboa, IST-Tagusparque, Porto Salvo, Portugal; 2 ATP-group, Porto Salvo, Portugal; 3 Centro de Biologia Molecular e Ambiental, Universidade do Minho, Braga, Portugal; 4 Departamento de Matemática e Aplicações, Universidade do Minho, Braga, Portugal; Tianjin University of Technology, CHINA

## Abstract

From work contracts and group buying platforms to political coalitions and international climate and economical summits, often individuals assemble in groups that must collectively reach decisions that may favor each part unequally. Here we quantify to which extent our network ties promote the evolution of collective fairness in group interactions, modeled by means of Multiplayer Ultimatum Games (**MUG**). We show that a single topological feature of social networks—which we call *structural power*—has a profound impact on the tendency of individuals to take decisions that favor each part equally. Increased fair outcomes are attained whenever *structural power* is high, such that the networks that tie individuals allow them to meet the same partners in different groups, thus providing the opportunity to strongly influence each other. On the other hand, the absence of such close peer-influence relationships dismisses any positive effect created by the network. Interestingly, we show that increasing the *structural power* of a network leads to the appearance of well-defined modules—as found in human social networks that often exhibit community structure—providing an interaction environment that maximizes collective fairness.

## Introduction

The human predisposition to be fair shapes decision-making and drives the outcome of social interactions [1–6]. The influence of fairness is often strong enough to overcome rationality and selfishness, posing a challenge to mathematical models that aim to incorporate the complexity of human interaction and thus justify fair behavior. Factors such as the cultural setting [7], community size, engagement in large-scale institutions [8], or even the socio-economic segment of the individuals [9], may provide clues regarding the propensity to be fair. Concerns about fairness may even lead individuals to decide, collectively, to give up some of their wealth to punish unfair behavior of others [10]. For instance, in the collective bargaining of work contracts, recognized in international human rights conventions, one has groups of individuals with different interests, where the fairness level of the outcome is ultimately shaped by the collective decision of employees and employer(s). Another less formal example is found in the Chinese concept of *tuangou*, where a group of people approaches a seller, offering to buy a large amount of items and negotiating reduced prices [11]. Today, *tuangou* provides a metaphor of many (collective) group buying platforms that aggregate millions of users in huge social networks [11–13]. Collective fairness decisions are also part of the process of policymaking by coalitions [14]. Political coalitions constitute decision units prevalent in a myriad of institutional settings (from parliamentary democracies to authoritarian regimes with power being divided among entities that legitimate the authority [14]), and their policies are only effective if the coalition members support or subordinate to the proposals made, which may favor each part unequally. In fact, from international climate and economic summits down to routine daily life arguing about the preferred restaurant to schedule a group dinner, many more examples could be added, all with a common backbone: interactions take place in groups in which individual assessment of fairness contributes to the overall degree of fairness reflected in the (collective) group decision process.

While the dynamics of fairness in two-person interactions has been given significant attention, mostly in the context of Ultimatum Games (**UG**) [4, 5, 15–21], the challenges posed by groups and associated fairness of collective decisions have not received corresponding emphasis. Furthermore, the fact that individuals often participate in multiple groups makes it important to understand to which extent the interplay between individual decision and participation in multiple groups (where collective action is at stake) influences overall fairness. To address this issue, we investigate the population dynamics arising from a Multiplayer Ultimatum Game (**MUG**), where proposals are made to groups [22] here defined by an underlying network of contacts [23–29]. We conclude that different networks lead to variable degrees of global fairness. In particular, we define a new network property, that we call Structural Power (**SP**, further detailed in Methods), that measures the prevalence of one individual (A) in the interaction groups of another (B) (normalized as the fraction of interaction groups of B where A also takes part). We show that this metric is instrumental and sufficient to identify those networks that maximize fairness at a global, population-wide level.

While in the 2-player **UG** a Proposer decides how to divide a given resource with a Responder and the game only yields payoff to the participants if the Responder accepts the proposal [3], in the N-player **MUG** proposals are made by one individual (the Proposer) to the remaining *N*-1 Responders, who must individually reject or accept the proposal [22]. Since individuals may act both as Proposers and Responders, we shall assume that each individual has a strategy characterized by two real numbers, *p* and *q*. The Proposer will try to split the endowment, offering *p* to the Responders. Each of the Responders will individually accept the offer made to the extent that his/her *q-*value is not larger than the *p*-value of the Proposer. Overall group acceptance will depend upon *M*, the minimum fraction of Responders that must accept the offer before it is valid. Consequently, if the fraction of individual acceptances stands below *M*, the offer will be rejected. Otherwise, the offer will be accepted. In this case, the Proposer will keep 1-*p* to himself and the group will share the remainder, that is, each Responder gets *p/*(*N*-1). If the proposal is rejected, no one earns anything [22]. The individual fitness arising from a specific group stands as the accumulated payoff obtained after individuals engage in *N* instances of the game, where each individual of a group acts once as a Proposer, and *N*-1 times as a Responder. In each instance, a fair split will be characterized by *p* = 1-1/*N*, as in this case both Proposer and Responders will get the same fraction of the offer. Empathy means that *p = q*, i.e., one offers precisely what one is willing to accept.

**Fig 1:**
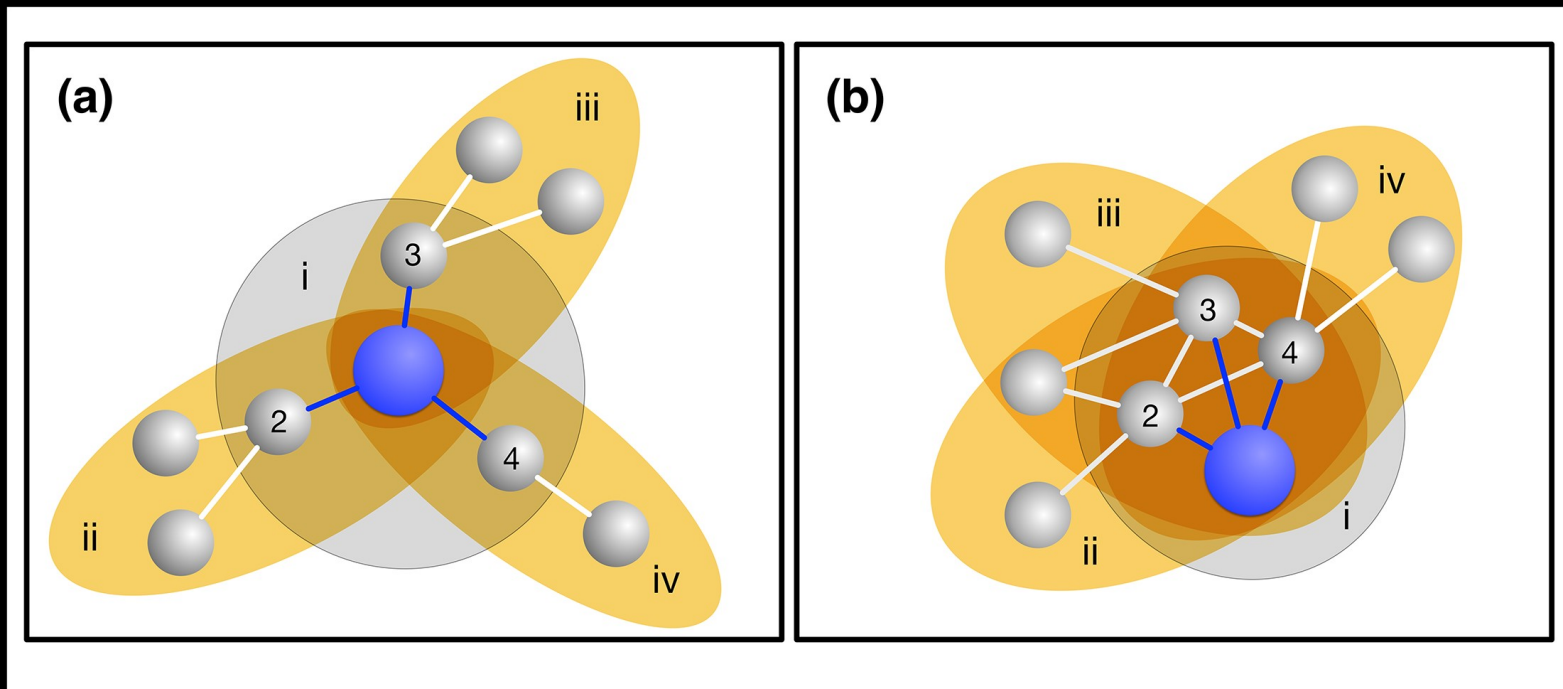
Examples of group formation. We represent all the groups where a focal individual, positioned in the blue node, participates. In both **(a)** and **(b)**, the focal individual has a connectivity of 3 (blue links) thereby playing in 4 different groups: one centered on herself (represented by a grey circle) and the 3 others centered on her (numbered) neighbors (represented by yellow ellipses). For instance, the groups represented by the ellipses *iv* contain all neighbors of individuals with number 4 (including the focal individual). The motifs presented in **(a)** and **(b)** differ in the overlap of the groups where the blue nodes take part, and consequently, the influence that those neighbors exert and are subject to. In **(a)**, none of the 1^st^ neighbors of the focal individual are 1^st^ neighbors of each other; thus, the focal individual only meets each of her/his neighbors in two groups. In **(b)**, all 1^st^ neighbors of the focal individual are directly connected, which means that individuals 2, 3 and 4 take part in all the groups where the focal individual also takes part. Thus, they influence each-other more in **(b)** than in **(a)**. The structural power (**SP**, defined in Methods and based on the prevalence of one individual in the interaction groups of another), provides a quantitative measure of the influence capacity of any node onto another (see Methods where we show that the influence extends to second neighbors). When applied to the entire network, the **SP** is thus higher in **(b)** than in **(a)**.

As detailed in Methods, we start from a population of size *Z*, much larger than the group size *N*, and equip individuals with values of *p* and *q* drawn from a discretized uniform probability distribution in the interval [0,1] containing 101 values (discretized to the closer multiple of 0.01). As already mentioned, to model the interplay between different interaction group assortments, we assume that individuals in the population are arranged in a graph (or network). In line with previous studies [25–27], each neighborhood defines a group, whereas the fitness *F*_*i*_ of an individual *i* of degree *k* is determined by the payoffs resulting from the game instances occurring in *k+*1 groups: one centered on her neighborhood plus *k* others centered on each of her *k* neighbors. In other words, each node with degree *k* defines a group with size *N = k+*1, including that node (*focal*) and the neighbors. Fig 1 provides pictorial representations of this group formation process. In homogeneous populations, every individual participates in the same number of groups (and **MUG** instances), all with the same size. Often, however, individuals face different numbers of collective dilemmas (depending, e.g., on their social position) that may also have different sizes. Such a dimension of social diversity is introduced here (Fig 4) by considering heterogeneous networks [30].

Social success drives the evolution of strategies in the population, that is, we implement strategy revision by social learning [26, 31–35], assuming that the behavior of individuals that perform better (i.e. achieve higher fitness) will spread faster in the population as they will be imitated with higher probability (see Methods for details).

We assume that individuals do not have direct access to the set of rules that define the behavior of others—instead, they perceive their actions, and therefore, errors of perception may be relevant. Consequently, whenever a pair (*p*,*q*) is copied, the final value will be perturbed by a random shift uniformly drawn from the interval [*-ε*,*ε*], reflecting the myopic nature of the imitation process. This process occurs along the social ties defined by the underling network [25].

## Results and discussion

**Fig 2:**
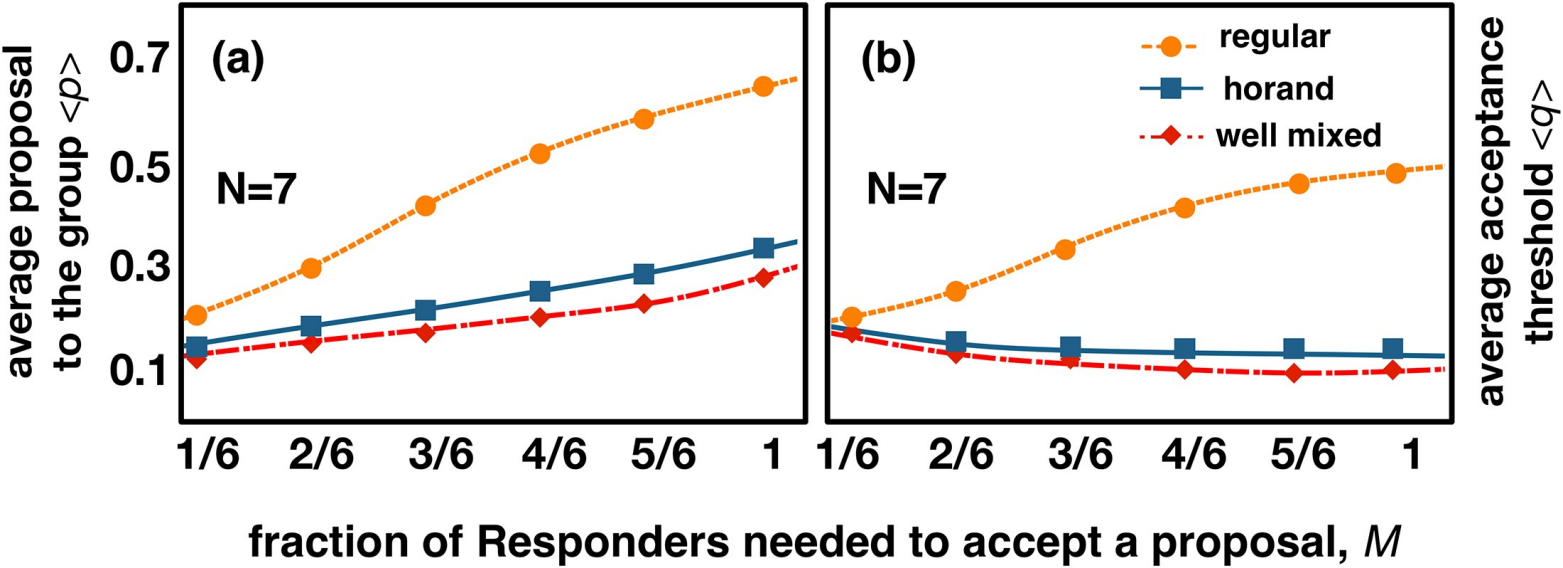
Average values of proposals and acceptance values that emerge for different topologies. The average values of the **(a)** proposals, <*p>* and **(b)** acceptance thresholds, <*q>*, as a function of the threshold *M* **(**the fraction of individual acceptances needed to ratify a proposal in **MUG)**, when **MUG** is played on unstructured populations (*well-mixed*), on regular rings (*regular*) or on random networks with homogeneous degree distribution (homogeneous random, *horand*, generated by swapping the edges initially forming a ring [37, 40, 66]). *M* has a positive effect on the average values of <*p*> [22]. Notwithstanding, this effect is much more pronounced in the case of regular networks, where we also witness a similar increase in the average values of <*q*>. Other parameters: average degree *<k> =* 6 (meaning that groups have a constant size of *N* = 7); population size, *Z* = 1000; mutation rate, *μ* = 0.001; imitation error, *ε* = 0.05 and selection strength, *β* = 10 (see Methods for definitions of all these parameters).

We start by simulating **MUG** on regular rings (*regular*) [36], and in homogeneous random networks (*horand*) [37] (see Methods for information regarding the construction and characterization of both networks, together with details of the simulation procedures). As Fig 2 shows, regular networks induce higher fairness and empathy, when compared with homogeneous random networks. Furthermore, there is an increase with *M* in both <*p*> and <*q*>, unlike what is observed for the other 2 classes of networks.

**Fig 3:**
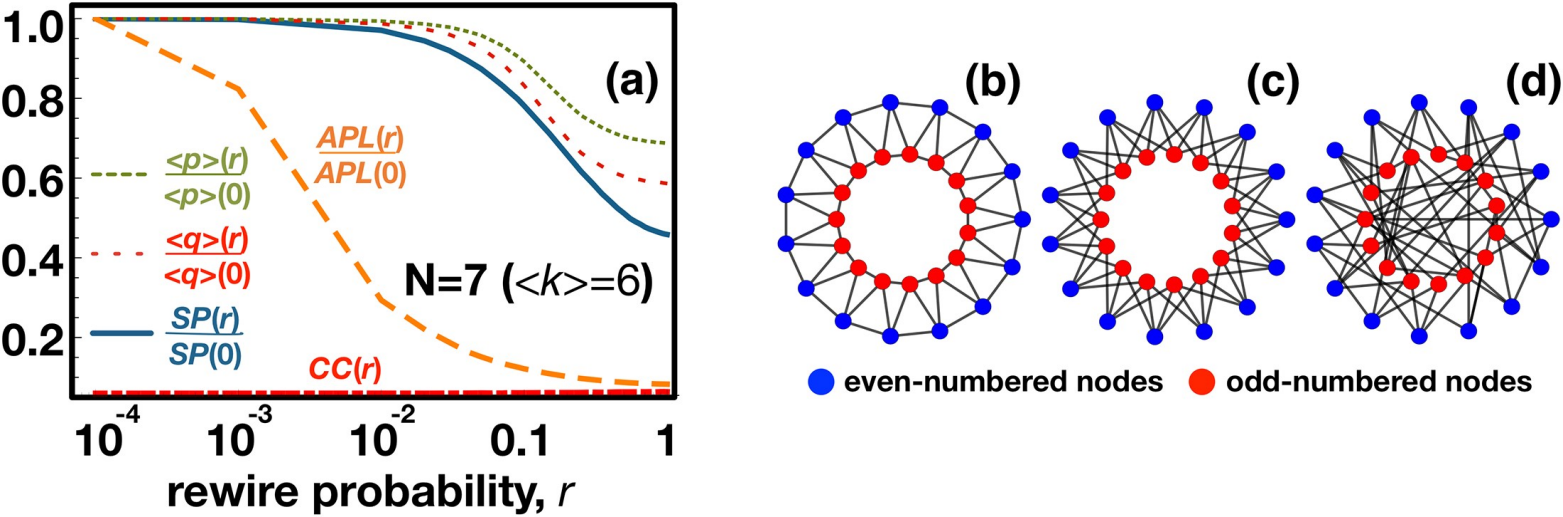
Impact of structural power on fair collective action. We interpolate between a regular triangle-free ring (high **SP**, *r* = 0, panel **c**) and a homogeneous random graph (*r* = 1, low **SP**, panel **d**) by rewiring a fraction *r* of all edges in the network while keeping the degree distribution unchanged. Our starting topology (*r* = 0) differs from the conventional regular rings (illustrated, for comparison, in panel **b**) as, by construction, it avoids the creation of triangles, leading to a **CC** = 0. Panel **a**) shows how different global network properties change as we change *r* (note that in this case networks have <*k*> = 6, corresponding to group size *N* = 7) and, importantly, how they correlate with properties emerging from playing the **MUG** on these networks: besides the average values of offer, <*p>*, and acceptance threshold, <*q>*, we also depict the dependence of **CC**, **APL** and **SP**. Whereas the value of **CC** remains negligible for all *r*, (growing from 0 at *r =* 0 to 0.003 at *r* = 1) the dependence of <*p>* and <*q>* is fully correlated with that of **SP** and with none of the other variables plotted. Other parameters (see Methods): *M* = 0.5, *Z* = 1000, *<k>* = 6, *μ* = 0.001, *ε* = 0.05 and *β* = 10.

Despite the fact that both classes of networks exhibit the same Degree Distribution (**DD**), they have quite different Clustering Coefficients (**CC**) and also Average Path Lengths (**APL**) [36, 37]. The regular ring networks warrant a high **CC** which, in turn, ensures that individuals appear repeatedly in the interaction groups of others. The prevalence of a given individual in the interaction groups of another may be understood as a power relation [15, 38, 39], that is, as a measure of the influence that an individual *A* has in the goals (here, fitness) of another individual *B*. This influence is enhanced by the fraction of interaction groups of *B* in which *A* appears (see Methods). To further characterize this property, we define an explicit quantity, that we call the Structural Power (**SP**). At the individual level, the structural power of an individual *A* over another individual *B* is given by the fraction of all groups in which *B* participates that also include *A*. This quantity, conveniently normalized between 0 and 1, is further extended to define the (average) **SP** of a node in a network, as well as the (average) **SP** of an entire network. Full details are provided in Methods. It is important to point out, however, that **SP** and **CC** convey different properties of a network: For instance, whereas **CC** only accounts for the triangular motifs present in a network, the computation of **SP** also reflects existing square motifs. To isolate the effect of **SP** from **CC**—and also from **APL** and **DD**—we calculate the average proposals <*p>* and average acceptance threshold <*q>* emerging when **MUG** is played in a class of networks in which **CC** always remains close to 0, but **SP** is not negligible (see Fig 3 and Methods).

In particular, we interpolate between two low **CC** networks: *i*) A triangle-free regular ring (which can also be interpreted as a regular bipartite graph, with links connecting odd-numbered nodes to even-numbered nodes, exhibiting a high **SP**, Fig 3c) and *ii*) a homogeneous random graph (low **SP**, Fig 3d), obtained by randomly rewiring the links of the triangle-free network (see Methods). The interpolation is implemented by means of a parameter *r* defining the fraction of links to be randomly rewired. The procedure keeps the **DD** unchanged, as pairs of links are swapped during the rewire process [37, 40]. As we depict in Fig 3, irrespectively of **CC**, **APL** and **DD**, the dependence of both *<p>* and *<q>* is fully correlated with that of **SP** and not with any of the other quantities.

**Fig 4:**
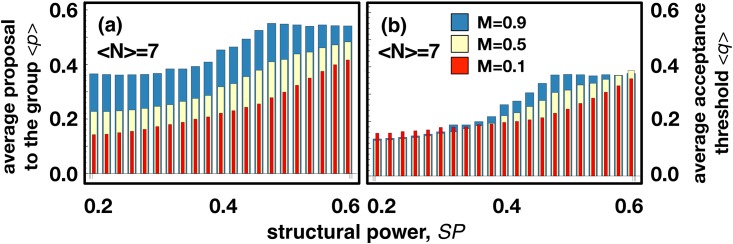
The effect of network SP on fairness. (**a**) Average proposals *<p>*, and (**b**) average acceptance thresholds <*q>*, when **MUG** is played in structured populations with different values of average **SP**. *M* stands for the fraction of individual acceptances needed to ratify a proposal in **MUG.** *M =* 0.5 means that, at least, half of the Responders have to accept the proposal, in order for it to be ratified by the group and have a positive effect on payoffs. When the game is played in networks with increased **SP**, the final values of <*p>* and <*q>* increase, i.e., strategies evolve to fairer levels. Also, for 0.4<**SP**<0.58 (covering the regular networks analyzed in Fig 1, with **SP** = 0.5), an increase in *M* also leads to an increase in <q>. Other model parameters: average degree, *<k> =* 6 (which means that groups have an average size of *<N>* = 7); population size, *Z* = 1000; mutation rate, *μ* = 0.001; imitation error, *ε* = 0.05 and selection strength, *β* = 10.

To further analyze the impact of **SP** on the levels of fairness, we design networks with different **SP** by optimizing the link structure of a random network until a desired pre-defined **SP** is achieved (see Methods). Fig 4 shows the average proposal (*<p>*), and acceptance threshold (*<q>*) we obtain, now as a function of the network **SP**. Clearly, high values of **SP** lead to higher values of <*p>* and <*q>*, in which case individuals adopt fairer strategies.

**Fig 5:**
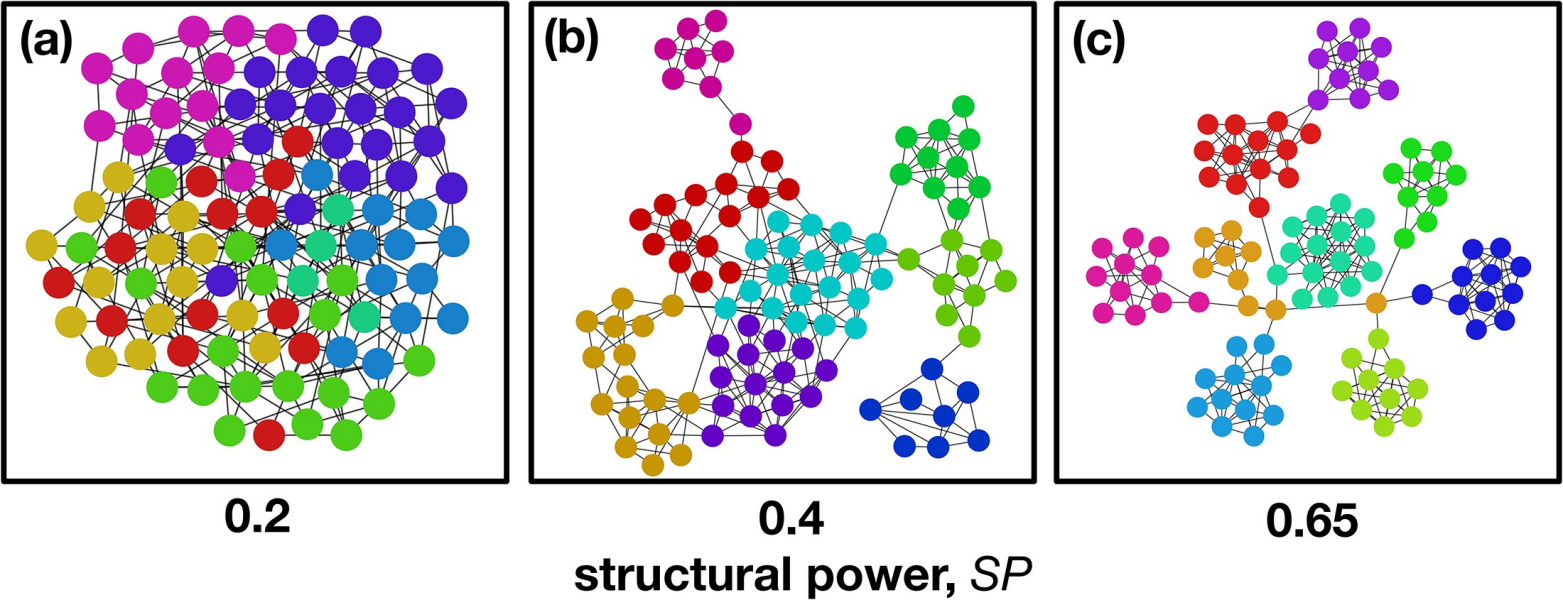
Intuitive representation of graphs with different average SP. The Fig provides an intuition for the effect of increasing **SP** in a small network of 100 nodes, while keeping the average degree, *<k>* = 6, constant. As the **SP** increases ((**a**) **SP** = 0.2, (**b**) **SP** = 0.4 and (**c**) **SP** = 0.65), different modular sub-structures increasingly appear. The disposition of nodes follows the Force Atlas algorithm [78] and the color scheme represents the detected communities by the Louvain method [78, 79].

Fig 5 illustrates the structural effects induced by maximizing the **SP** of a network, while keeping the average degree <*k*> constant. Additionally, we concentrate our analysis on sparse structures (<*k*> << Z), as it is often the case in social networks [41, 42]. When maximizing the **SP** under these constraints, one witnesses the emergence of highly modular sub-structures, with the concomitant appearance of different communities [43]. In fact, each node acquires high **SP** by repeatedly appearing in the interaction groups of individuals belonging to the same community, which leads, as a consequence, to a distinguishing characteristic of modular networks: high average **SP**.

The prevalence of fairness in small communities where members share a large number of connections fits well with the empirical studies that argue for the trust and confidence provided by this kind of community structures [44–47]: All the individuals of the community monitor the interactions occurring within links, referred as *strong ties* or *embedded* links. In fact, it is worth noting how easier is to render punishment effective (where punishment here means having a high acceptance threshold, *q*, thus rejecting low proposals and preventing unfair Proposers to earn payoff) when networks exhibit high **SP** and, therefore, many local communities: In such cases one punisher may be enough to derail all the low proposals of one unfair neighbor. On the other hand, in structures with low **SP**, the fact that each unfair individual plays in several groups with different opponents requires different punishers to be present in those groups, in order for unfairness to be effectively penalized. This way, communities provide the needed lever to trigger fair strategies. Naturally, explicit forms of sanctioning could be devised, such that its impact, together with its relation with particular network topologies, can be extended to other social dilemmas and interaction contexts [10, 17, 48–51]. In particular, we conjecture that networks with high **SP** should play an increased role when working as interacting structures for multiplayer games with thresholds [26, 27, 52, 53].

Additionally, networks with high **SP**, besides providing the right context for preventing unfair proposals, can also confer a relative advantage to individuals prone to reject low offers and make fair proposals. Having one individual with high **SP** and high acceptance threshold often implies that the only proposal accepted in the neighborhood is precisely her/his own. Naturally, this can only happen if that individual is able to take part in a large fraction of his/her peers, which, again, translates into a high **SP**.

Here we investigate fairness based on group decisions by means of an evolutionary game theoretical model employing **MUG**, played along the links of complex social networks. Our results show that the **SP** of a network constitutes a key observable indicating the feasibility that fairness emerges in the population, in both regular (Figs 2 and 3) and heterogeneous networks (Fig 4) and in situations where resorting to well-established quantities such as **CC** can be elusive (Fig 3).

Finally, this work can be related with the important concept of governance by means of polycentric sanctioning institutions [52, 54]. To this end, let us assume that every Responder conceals a potential punisher and each group where **MUG** is played constitutes a center of decision. This perspective re-positions the present model into an interestingly polycentric perspective, as now Responders with high **SP** spawn many overlapping interaction groups which, in turn, can be related to the problem of interdependence between groups. As mentioned by V. Ostrom “*Polycentric connotes many centers of decision making that are formally independent of each other*. *Whether they actually function independently*, *or instead constitute an interdependent system of relations*, *is an empirical question in particular cases*” [54, 55]. We find that the question of the interdependence of groups taking part in collective decisions, here quantified by means of the **SP**, may be central in promoting seemingly paradoxical human features such as fairness.

## Methods

### Game, payoff and fitness

Following the conventional notation of **UG** [17], the total amount initially given to the *Proposer* playing **MUG** is equal to 1. In a group of *N* individuals, the proposal made is *p* ∊ [0,1] and each of the *N*-1 *Responders* has an acceptance threshold *q* ∊ [0,1]. Once the proposal is made, each *Responder* will individually state his acceptance (if *q*≤*p*) or rejection (if *q*>*p*). Overall, the group acceptance depends upon a minimum fraction of individual acceptances, *M*. This can be summarized in a variable *a*_*i*_, assuming the value 1 if the proposal by individual *i* is accepted, and 0 otherwise [22]:

$$a_{i}=\{\begin{array}{l} 1\;,\text{ if }\Sigma_{j=1,j\neq i}^{N}\Theta (p_{i}-q_{j})/(N-1)\geq M\\ 0\;,\text{ otherwise} \end{array}$$ (1)

where Θ(*x*) is the Heaviside function, assuming the value 0 when *x*<0 and 1 otherwise. The payoff Π_i_ earned by an individual *i* in a group of *N* individuals, will be given by adding the result of acting once as the *Proposer*—Π_*P*_ = (1 − *p*_*i*_)*a*_*i*_—and *N*-1 times as a *Responder*— $\Pi_{R}=\frac{1}{N-1}\sum_{k=1,k\neq i}^{N}p_{k}a_{k}$, where *p*_*k*_ is the offer of individual *k* and *a*_*k*_ refers to the proposal of individual *k*. It is worth noting that the maximum payoff of an individual *i* is obtained when *p*_*i*_ is the smallest possible and all other *p*_*k*_ (the offers of opponents) are maximized. Therefore, there is a high pressure to *free-ride*, that is, offering less and expecting that others will contribute. Furthermore, dividing the game in two stages and reasoning in a backward fashion, the conclusions regarding the *sub-game perfect equilibrium* of this game anticipate the use of the smallest possible *p*_*i*_ and *q*_*i*_, irrespectively of *N* and *M* [56], mimicking the conclusions for the traditional 2-person **UG** [57]. The fitness is given by the accumulated payoff earned after playing in all possible groups.

### Networks

An underlying network of contacts defines the groups in which individuals play. One node (*focal*) and its direct neighbors define a group. An individual placed in a node with connectivity *k* will play in *k*+1 different groups. In Fig 1 we provide intuitive representations for this group formation process (where the structural power **SP** is defined next). We use four classes of networks, namely, *i)* regular rings [36], *ii)* regular triangle-free rings, *iii)* homogeneous random networks [37] and *iv)* networks with pre-defined average **SP**. Regular rings, with degree *k*, are traditionally constructed by *i*) creating a numbered list of nodes and *ii*) connecting each node to the *k* nearest neighbours in that list [36]. Similarly, we generate regular triangle-free rings (with degree *k*) by connecting one node (source) with the closest *k* nodes, yet only those at an odd distance (in the list) to the source (in the language of graph theory, this corresponds to define a (*k*,*k*)-biregular graph using the odd-numbered and even-numbered nodes as disjoints sets). This allows preventing the occurrence of triangles (i.e., adjacent nodes of a given node that are, themselves, connected) which would contribute to increase **CC**. In Fig 3, we interpolate between a regular triangle-free ring and a homogeneous random graph following the algorithm proposed in [37]. We introduce a parameter *r* which gives the fraction of edges to be randomly rewired: for *r* = 0 we have a regular triangle-free ring, whereas for *r* = 1 all edges are randomly rewired and we obtain a homogeneous random graph. We adopt a rewiring mechanism which does not change the degree distribution [37, 40]. The algorithm resumes to repeat the following two-step circular procedure until a fraction *r* of all edges are successfully rewired: *1)* choose—randomly and independently—two different edges which have not been used yet in step 2, and *2)* swap the ends of the two edges if no duplicate connections arise. In Fig 4, to generate networks with pre-defined average **SP**, we apply an optimization algorithm to a random network. The random networks are generated by rewiring all the edges of regular ring [36]. Let us now assume that we want to build a network with average **SP** equal to *sp*_*max*_. We re-organize the link structure of the initial network using a stochastic multi-step process such that, in each step, an edge of network is rewired at random (with no repeated edges allowed). The move is accepted if two criteria are met: *1)* the resulting network remains connected and *2)* the average **SP** of the resulting network (*sp*_*t*_) increases (compared to the previous value) or passes the following stochastic criterion: a move in which **SP** decreases is accepted with probability *λ(sp*_*max*_*-sp*_*t*_*)*, where *λ* controls the probability of accepting an erroneous move. That means that the probability of accepting a rewire that decreases **SP** is lower as we get close to the desired **SP**. This is an optimization feature similar in spirit to the well-known *simulated annealing* [58]. We used *λ =* 0.001.

### Structural Power (SP)

The population structure provides the definition of the different groups of interaction, which may overlap to variable extent [59]. Considering the usual group formation that we address (in which one node defines, together with his/her direct neighbors, a group), individuals may appear repeatedly in the interaction groups of others. As said, this repetition may provide increased **SP** to some individuals over others.

We define the **SP** of *A* over *B* as $SP_{A,B}=\frac{\left|I(A)\cap I(B)\right|}{\left|I(B)\right|}$, where *I*(*X*) represents the groups in which individual *X* appears and |*I*(*X*)*|* represents the number of groups in *I*(*X*). One may note that, using the Kronecker *δ*_*A*,*B*_ to identify edges between *A* and *B* (e.g, 1 if an edge connects nodes *A* and *B* and 0 otherwise), and denoting by *o*_*A*,*B*_ (overlap) the number of common neighbors of A and B and by *k*_*X*_ the number of neighbors of *X*, then the **SP** of *A* over *B* is given by $SP_{A,B}=\frac{2\delta_{A,B}+o_{A,B}}{k_{B}+1}=\frac{2\delta_{A,B}+\sum_{i\in nodes}\delta_{A,i}\times \delta_{i,B}}{\sum_{i\in nodes}\delta_{i,B}+1}$.

Intuitively, if one individual is a direct neighbor of other (*δ*_*A*,*B*_ *=* 1), they will meet in at least two groups, where each one will be the *focal* in each group. They will meet again if they have a common neighbor *i* (*δ*_*A*,*i*_ × *δ*_*i*,*B*_ = 1), and thus whenever *A* and *B* are direct neighbors, *o*_*A*,*B*_ counts the number of triangular motifs involving both *A* and *B*. If *B* has connectivity *k*_*B*_, then this node participates in *k*_*B*_+1 groups, providing the proper normalization to *SP*_*A*,*B*_. Importantly, even if *A* and *B* are not direct neighbors, *SP*_*A*,*B*_ will not be zero, in general (e.g., square motifs may lead to *o*_*A*,*B*_≠0).

The average **SP** of one node is defined as $SP_{A}={\left|R(A)\right|}^{-1}\sum_{i\in R(A)}^{}SP_{A,i}$, where *R(A)* is the set of individuals reached by individual *A*, either directly or through a common neighbor, and |*R*(*A*)| is the size of this set. Finally, the average **SP** of one network is the average **SP** taken over all of its nodes. As an example, in Table 1 we show the average structural power (**SP**) of several social networks [60] including a sample of Facebook [61], an email communication network (Enron email network, in which nodes are email addresses and edges represent at least one email sent between addresses [62, 63]), and several collaboration networks inferred from the co-authorship of papers on arXiv [64], in topics such as General Relativity (GrQc), High Energy Physics Phenomenology (HepPh), High Energy Physics Theory (HepTh), Astrophysics (AstroPH) or Condensed Matter (CondMat). Interestingly, all the abovementioned networks show a global **SP** significantly higher that the one obtained from a random network [36, 65] with the same size (*Z*) and average degree (<*k>*) (see **SP**_rand_1). A similar result is obtained if, instead, we compare the **SP** of empirical networks with randomized networks of the same *Z*, <*k>*, and degree distributions (**SP**_**rand**_**2**). Following refs. [37, 40, 66], **SP**_**rand**_**2** was computed as the **SP** of the network that results from swapping random pairs of edges for 10*Z*<*k>* times.

**Table 1:** SP of different networks. See Methods for details.

Dataset	Z	<k>	SP	SP_rand_1	SP_rand_2
**Facebook**	4039	44	0.14	0.05	0.04
**Email**	36692	10	0.25	0.10	0.15
**AstroPH**	18772	21	0.15	0.05	0.05
**CondMat**	23133	8	0.26	0.12	0.12
**GrQc**	5242	6	0.41	0.17	0.20
**HepPh**	12008	20	0.22	0.05	0.08
**HepTh**	9877	5	0.33	0.24	0.20

### Evolutionary dynamics in structured populations

Instead of revising their strategies through rational reasoning, humans often resort to the experiences and successes of others to select their next move, as, in fact, has been shown to be the case in the context of public donations [67–69]. Such an interacting dynamical process, grounded on peer-influence and imitation, creates a behavioral ecosystem in which strategies and behaviors evolve in time, whereas the returns of each individual depend on the actual frequency of each strategy present in its neighborhood. Fitness is said to be *context-dependent*. Here we adopt such social learning dynamics [17, 23, 25–27, 31–35, 70, 71], which is also well suited to be used in the framework of evolutionary game theory. The baseline assumption is that individuals performing better when playing **MUG** (i.e. those achieving higher accumulated payoffs) will be imitated more often and thus their strategies will spread in the population. Social success drives the adoption of strategies in the population. Imitation occurs by copying behavior through the social ties, statically defined by the underlying network.

### Simulations

Numerical results were obtained for structured populations of size *Z* = 1000. Simulations take place for 50000 generations, considering that, in each generation, all the individuals have the opportunity to revise their strategy through imitation. At every (discrete and asynchronous) time step, two individuals *A* and *B* (neighbors) are randomly selected from the population and their individual fitness is computed as the accumulated payoff in all possible groups, provided by the underlying structure; subsequently, *A* copies the strategy of *B* with a probability χ that is a monotonic increasing function of the fitness difference *f*_*B*_*-f*_*A*_, following the pairwise comparison update rule [72]— $\chi ={\left(1+e^{-\beta (f_{B}-f_{A})}\right)}^{-1}$. The parameter *β* conveniently specifies the selection pressure (*β =* 0 represents neutral drift and *β*→∞ represents a purely deterministic imitation dynamics). Additionally, imitation is myopic: The copied *p* and *q* values will suffer a perturbation due to errors in perception, such that the new parameters will be given by *p*' = *p* + *ξ*_*p*_(*ε*) and *q*' = *q* + *ξ*_*q*_(*ε*), where *ξ*_*p*_(*ε*) and *ξ*_*q*_(*ε*) are uniformly distributed random variables drawn from the interval [*-ε*,*ε*]. This feature not only *i)* models a slight blur in perception but also *ii)* helps to avoid the random extinction of strategies, and *iii)* ensures a complete exploration of the strategy spectrum, given that the pairwise comparison does not introduce new strategies in the population [73]. To guarantee that *p’* and *q’* are not lower than 0 or higher than 1, we implement reflecting boundaries at 0 and 1, e.g., if *p’>*1 then *p’* is set to 2*-p’* [73–75]. Furthermore, with probability *μ*, imitation will not occur and the individual will adopt random values of *p* and *q*, proceeding through a random exploration of behaviors. We use *μ* = 1/Z throughout this work. The effect of varying this parameter is similar to the one verified when changing *ε*: an overall increase of randomness leads to higher chances of fairer offers [22, 76, 77]. For each combination of parameters, the simulations were repeated 100 times (using 10 different networks from each class studied), whereas each simulation starts from a population where individuals are assigned random values of *p* and *q* drawn uniformly from an evenly discretized strategy space in the interval [0,1] containing 101 values. The average values of *p* and *q* obtained, denoted by *<p>* and *<q>*, are both a time and ensemble average, taken over all the runs and considering the last 25*%* of generations, disregarding the initial transient period.
